# Mineralocorticoid Receptor and Aldosterone-Related Biomarkers of End-Organ Damage in Cardiometabolic Disease

**DOI:** 10.3390/biom8030096

**Published:** 2018-09-18

**Authors:** Stefania Gorini, Vincenzo Marzolla, Caterina Mammi, Andrea Armani, Massimiliano Caprio

**Affiliations:** 1 Laboratory of Cardiovascular Endocrinology, IRCCS San Raffaele Pisana, Via di Val Cannuta 247, 00166 Rome, Italy; stefania.gorini@sanraffaele.it (S.G.); vincenzo.marzolla@sanraffaele.it (V.M.); caterina.mammi@sanraffaele.it (C.M.); andrea.armani@sanraffaele.it (A.A.); 2 Department of Human Sciences and Promotion of the Quality of Life, San Raffaele Roma Open University, 00166 Rome, Italy

**Keywords:** mineralocorticoid receptor, aldosterone, PBMC, NGAL, Gal-3, PTGDS, adipose tissue

## Abstract

The mineralocorticoid receptor (MR) was first identified as a blood pressure regulator, modulating renal sodium handling in response to its principal ligand aldosterone. The mineralocorticoid receptor is also expressed in many tissues other than the kidney, such as adipose tissue, heart and vasculature. Recent studies have shown that MR plays a relevant role in the control of cardiovascular and metabolic function, as well as in adipogenesis. Dysregulation of aldosterone/MR signaling represents an important cause of disease as high plasma levels of aldosterone are associated with hypertension, obesity and increased cardiovascular risk. Aldosterone displays powerful vascular effects and acts as a potent pro-fibrotic agent in cardiovascular remodeling. Mineralocorticoid receptor activation regulates genes involved in vascular and cardiac fibrosis, calcification and inflammation. This review focuses on the role of novel potential biomarkers related to aldosterone/MR system that could help identify cardiovascular and metabolic detrimental conditions, as a result of altered MR activation. Specifically, we discuss: (1) how MR signaling regulates the number and function of different subpopulations of circulating and intra-tissue immune cells; (2) the role of aldosterone/MR system in mediating cardiometabolic diseases induced by obesity; and (3) the role of several MR downstream molecules as novel potential biomarkers of cardiometabolic diseases, end-organ damage and rehabilitation outcome.

## 1. Introduction

The mineralocorticoid receptor (MR) is a member of the nuclear receptor family and acts as a ligand-dependent transcription factor. It was initially identified to regulate blood pressure through its ability to modulate renal sodium handling in response to aldosterone [1,2,3]. Importantly, aldosterone is not the exclusive ligand of MR. Cortisol and aldosterone display similar affinity and specificity for the MR [4]. In tissues with low 11β-hydroxysteroid dehydrogenase type 2 (11β-HSD2) activity, which converts cortisol to inactive cortisone, MR activation is mainly regulated by circulating glucocorticoids [5].

It is now evident that the MR is expressed in many tissues other than the kidney. Importantly, MR is expressed in the heart [6,7], in all cell types of the vasculature, including smooth muscle cells (SMCs), endothelial cells (ECs) and fibroblasts, and has also been found in adipose tissue [8]. In this context, the MR has a relevant role in the control of cardiovascular and metabolic function [9,10,11].

Dysregulation of the aldosterone/MR signaling has been identified as an important cause of several diseases. Indeed, high plasma levels of aldosterone are strictly associated with hypertension, obesity and increased cardiovascular risk [12]. Several studies demonstrated that obese and hypertensive patients display increased plasma and urinary levels of aldosterone [13,14,15]. Molecular mechanisms underlying vascular changes in hypertension are not completely understood, but a role for aldosterone has been suggested. Accumulating evidence has demonstrated that aldosterone displays powerful vascular effects and acts as a potent pro-fibrotic agent in cardiovascular remodeling [16,17]. Indeed, MR activation in human coronary artery SMCs regulates several genes involved in vascular fibrosis, calcification and inflammation, such as collagen types I and III, the parathyroid hormone receptors and interleukin (IL)-16 [16]. The MR is known to regulate genes involved in inflammation and oxidative stress in human coronary ECs [10]. Reactive oxygen species (ROS) have also been suggested to mediate the detrimental effects of aldosterone in the vasculature through MR activation [18,19].

Ligand-independent transcriptional activation of the MR has also been described, since MR can be activated under conditions of high oxidative stress, even without any increase in circulating agonists [20]. It is now clear that several molecules, other than aldosterone, can activate MR. For instance, Rac1 represents an important activator of MR. It is a small GTPase belonging to the Rho family and it is involved in the activation of MR in the kidney and in the heart [21,22,23]. Rac1 overexpression in cardiomyocytes of rats upregulates MR transcription [24]. Its overexpression in a mouse model of pressure overload-induced heart failure (HF) can increase MR protein and MR target genes expression in the heart [23]. Clinical evidence suggests that the interaction between Rac1 and MR plays a major role in cardiovascular damage induced by high sodium intake in humans as Rac1 expression positively correlates with MR expression under high sodium intake dietary regimens [25].

In addition to its classical genomic effects, aldosterone elicits rapid actions that do not require transcription or translation. These effects can be mediated by crosstalk of the MR with several membrane-associated signaling pathways, including transactivation of tyrosine kinases (i.e., epidermal growth factor receptor (EGFR), platelet-derived growth factor receptor (PDGFR) and insulin-like-growth factor 1 receptor (IGF1R) [26], or G protein coupled receptors. Among these, G protein-coupled estrogen receptor 1 (GPER1) has been proposed as a novel aldosterone receptor, even if a direct binding or interaction between GPER1 and aldosterone still awaits demonstration [27]. Such immediate effects are mostly involved in ion transport, but play also a relevant role in extrarenal tissues, contributing to the pathophysiological effects of MR and leading to inflammation, fibrosis and organ damage. Notably, genomic and nongenomic MR signaling interact closely, and their combined effect determines the long-term impact of altered MR activation at the level of vessels, heart and kidney [28,29,30]. This aspect was extensively reviewed by Ruhs et al. [26].

This review discusses the global pathophysiological relevance of aldosterone and MR-related pathways in cardiometabolic disease and obesity. In this context, we discuss the role of potential novel biomarkers related to the aldosterone/MR system, that could help identify early stages of end-organ damage (heart, vessels, kidney, and adipose tissue) in cardiometabolic diseases, as well as the outcome of therapeutic intervention and rehabilitation.

## 2. Cardio-Metabolic Effects of Altered Mineralocorticoid Receptor Activation

There is a large body of evidence that identifies the MR pathway as a valuable check-point for healthy or pathological cardiometabolic states. Indeed, an important role of MR activation in the pathogenesis of cardiometabolic diseases has been clarified [31]. Aldosterone, classically considered only as regulator of Na^+^ reabsorption, is known to trigger cardiovascular and renal tissue damage through different pathways, which are, at least in part, independent of its renal-mediated effects on blood pressure [10]. Indeed, extrarenal effects of aldosterone are relevant for production of extracellular matrix (ECM) components, and elicit several specific tissue responses, such as hypertrophy, remodeling and fibrosis, which are pathogenic and contribute to end-organ damage [32].

Notably, preclinical studies have shown that aldosterone causes end-organ tissue damage only in the context of an inappropriate salt status [33]. Indeed, pioneering studies clarified that aldosterone causes myocardial fibrosis only in rats maintained on a high-salt diet [34]. 

These pieces of evidence have also been observed in humans. In environmental conditions where the average daily intake of sodium is very low, physiologically circulating aldosterone levels can be very high, in response to sodium deficiency, but do not determine any deleterious cardiovascular or renal effects. It is therefore important to remark that, when aldosterone levels are out of the physiological feedback control loop, and become inappropriate for salt status, they can induce cardiovascular damage [12].

Primary aldosteronism (PA) is due to an autonomous overproduction of aldosterone by the adrenal gland, entirely unrelated to salt status [35]. This leads to a condition characterized by severe hypertension, low renin levels and severely increased cardiovascular risk, with a higher incidence of stroke, atrial fibrillation, and myocardial infarction [36]. Interestingly, chronic exposure to aldosterone in primary aldosteronism has been also associated to altered glucose homeostasis, and, in general, with a greater prevalence of the metabolic syndrome [37,38]. Fallo et al. described a higher prevalence of metabolic syndrome in patients with PA compared to those with essential hypertension [39]. In particular, altered glucose metabolism represented the best-established component of metabolic syndrome among PA patients. A possible reason for this observation relies on the inhibitory effects of aldosterone upon glucose-stimulated insulin secretion, as suggested by in vitro studies on isolated pancreatic islets [40,41]. In line with preclinical data, several clinical studies show that insulin sensitivity is reduced in PA patients compared to hypertensive controls [42,43,44].

Importantly, Catena et al. showed that PA is associated with insulin resistance and that pharmacological MR antagonism can reverse the insulin resistance status of primary aldosteronism patients [45].

Evaluation of longitudinal changes in metabolic risk factors in the Framingham offspring study showed that aldosterone is correlated to the development of metabolic syndrome. This correlation was also apparent in longitudinal changes in blood pressure and plasma levels of high-density lipoprotein (HDL) cholesterol, suggesting aldosterone and its associated pathways as potential biomarkers for metabolic risks [46].

Consequently, increased cardiovascular morbidity and mortality is related to several blood pressure-independent factors in patients affected by PA, such as cardiac myocardial fibrosis [47,48], cardiac remodeling [49], atherosclerosis with plaque rupture [50] and arrhythmias [51]. Moreover, several clinical studies reveal that circulating aldosterone levels are reliable predictors of cardiovascular ischemia [36,52]. Finally, strong evidence emerged from clinical trials in patients with heart failure and previous myocardial infarction that demonstrated MR pharmacological blockade protects from mortality and end-organ damage [53].

## 3. Contribution of Different Immune Cells Subsets to Aldosterone-Induced Inflammation

The state of hypertension induced by excessive secretion of aldosterone in PA patients is mostly due to the promotion of myocardial and vascular fibrosis [54,55]. However, a relevant role of oxidative stress [56], perivascular inflammation, and infiltration of T lymphocytes and antigen-presenting cells (APCs) in vessels has been also described [57,58,59]. Indeed, it is known that excessive production of aldosterone leads to hypertension by a pro-inflammatory state promoted by T cell immunity [57]. Pioneering studies by Selye et al. in 1949 showed that desoxycorticosterone (DOC), the first mineralocorticoid to be discovered, could induce a pro-inflammatory effect [60]. This finding was based on the observation that DOC was able to worsen clinical symptoms of rheumatoid arthritis, as well as induce a strong pro-inflammatory effect in animal models. In vitro and in vivo studies demonstrated that MR activation by aldosterone exerts its effects on vasculature and the heart, in part, by inducing an increase in oxidative stress [61].

In addition, aldosterone is able to induce vascular and cardiac inflammation through increased expression of inflammatory biomarkers, such as fibrinogen and plasminogen activator inhibitor-1 (PAI-1) [62,63]. Mineralocorticoid receptor activation in ECs is known to contribute to the induction of cardiac inflammation and remodeling by promoting the expression of vascular cell adhesion molecule 1 (VCAM1), as shown in animal models of hypertension [64]. Moreover, endothelial MR activation by aldosterone leads to the overexpression of the intracellular adhesion molecule-1 (ICAM-1) via an MR-responsive element in ICAM-1 promoter region [10,65,66], thereby enabling leukocyte adhesion to coronary artery ECs.

Activation of the MR by deoxycorticosterone acetate in the presence of high salt intake in mice represents a powerful model of hypertension and inflammation (DOCA-Salt model), which in turn leads to cardiovascular and renal fibrosis and cardiac remodeling [62,67]. Rickard et al. investigated the specific role of MR activation in ECs and studied vascular responses to aldosterone in EC-null MR (EC-MRKO) mice treated with DOCA-salt [68]. In the early stages (after eight days of treatment), macrophage infiltration and expression of myocardial proinflammatory genes (i.e., C-C chemokine receptor type 5 (CCR5) and inducible nitric oxide synthase (iNOS)) in EC-MRKO was prevented; mRNA levels of profibrotic genes in EC-MRKO mice (i.e., connective tissue growth factor (CTGF) and PAI-1) were significantly lower compared to wild type (WT) mice. Finally, CTGF expression and collagen deposition were significantly reduced in EC-MRKO mice. Reduced cardiac tissue macrophage infiltration determined the down-regulation of proinflammatory and profibrotic markers in the heart, along with a lower vascular expression of ICAM-1 and CTGF [68].

Interestingly, aldosterone amplifies its pro-inflammatory effects through the induction of osteopontin release in activated tissue macrophages and T-cells [69]. Recent studies also reveal that aldosterone induces renal tubulointerstitial inflammation/fibrosis and podocytes injury through the activation of the nucleotide-binding oligomerization domain (NOD)-like receptor family pyrin domain containing 3 (NLRP3) inflammasome, determining the expression of important inflammasome components, such as caspase 1, IL-1β and IL-18 [70,71]. Moreover eplerenone (a selective MR antagonist) is able to suppress the expression of critical inflammasome components, such as *Nlrp3* and *Caspase1*, in epididymal white adipose tissue (eWAT) and liver of obese mice. These data clearly show that MR represents a crucial player in the induction of inflammasome-mediated chronic inflammation in metabolic disorders [72]. 

**Figure 1:**
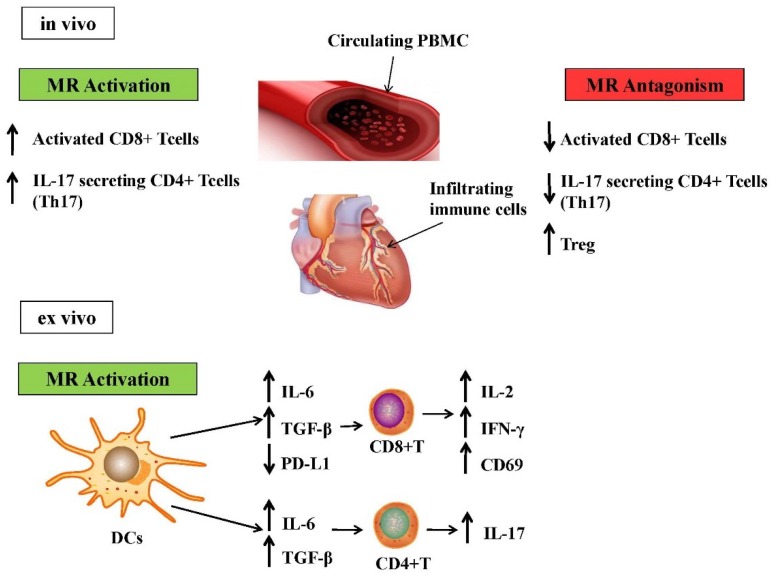
Effects of excess mineralocorticoid receptor (MR) activation on circulating and intra-tissue immune cells. Overactivation of MR upregulates CD8+ T cells and T helper 17 (Th17) cells in circulating peripheral blood mononuclear cells (PBMCs) and in immune cells infiltrating in the heart. On the other hand, MR antagonism is able to decrease Th17 polarization and to induce the T regulatory cell (Treg) phenotype. These cells subsets are primed by dendritic cells (DCs). Dendritic cells express MR and are induced by aldosterone to produce polarizing cytokines that are able to activate CD8+ T cells and to prime CD4+ T cells towards the Th17 phenotype. IL: interleukin; TGF-β: transforming growth factor-beta; PD-L1: programmed death-ligand 1; IFN-γ: interferon-gamma.

There is a large body of evidence showing that lymphocytes are important players in the development of chronic hypertension, perivascular mononuclear cell infiltration, and renal injury. In 1976, Svendsen observed that, upon DOCA-salt treatment, mice with normal thymus function and nude mice with genetical aplasia of the thymus, both displayed a significant increase in blood pressure after three weeks [73]. After 2–3 months, however, blood pressure increased and cell infiltration around intrarenal vessels was significantly more pronounced in WT than in nude mice, together with degenerative changes in the kidney, such as wedge-shaped infarcts. Thymus grafting in nude mice before DOCA-salt treatment recovered the ability of DOCA-salt treatment to induce chronic hypertension and intrarenal vascular disease, as previously seen in mice with normal thymus function [73]. More recent studies clarified how MR signaling is able to regulate the number of circulating T cells in human subjects and their homing to lymph nodes [74]. Under physiological conditions, T lymphocytes do not seem to contribute to systemic blood pressure, but DOCA-salt treatment, as well as angiotensin II (AngII) infusion, in hypertensive animal models, display an increase in intravascular and circulating T cells [57,58]. DOCA-salt or AngII infusion is also able to increase IL-17 secretion by T lymphocytes, as well as IL-17 protein in the heart and vessel wall [57,75]. Interestingly, recent studies on peripheral blood mononuclear cells (PBMCs) from hypertensive patients showed an increased prevalence of cytotoxic CD8+ T cells compared to normal subjects [76]. Accordingly, Amador et al. demonstrated the presence of CD8+ and IL-17+ T cells in PBMCs and splenocytes of hypertensive DOCA-salt-treated mice. Such effects were prevented by spironolactone, suggesting a role for the mineralocorticoid receptor. Moreover, spironolactone was able to decrease IL-17 expression and increase the synthesis of typical regulatory T cells (Treg) marker forkhead box P3 (FoxP3), indicating that MR blockade downregulates T helper 17 (Th17) and upregulates Treg cell polarization [77]. Li et al. recently demonstrated that pharmacological MR antagonism protects against cardiac dysfunction and hypertrophy induced by abdominal aortic constriction. Mineralocorticoid receptor antagonism decreased the accumulation and activation of CD4+ and CD8+ T cells in the murine heart. Moreover, T cell specific MR-knockout mice displayed reduced cardiac hypertrophy, fibrosis, and dysfunction after abdominal aortic constriction [78]. Interestingly, dendritic cells (DCs) express MR mRNA and protein, therefore they are able to respond to aldosterone [79]. Dendritic cells have the peculiar capacity to prime naive T cells (CD4+ and CD8+) modulating an adaptive immune response [80]. Herrada et al. demonstrated that aldosterone enhances CD8+ T cytotoxic cells activation in a DCs-dependent fashion [79]. Indeed, direct in vitro activation of T lymphocytes by aldosterone was not able to induce the overexpression of typical activation markers, such as IL-2 and CD69. On the other hand, pretreatment of DCs with aldosterone, followed by co-culture with purified T cells, determined the activation of CD8+ T cells, as shown by IL-2 and interferon-gamma (IFN-γ) secretion and CD69 upregulation, and CD4+ T lymphocytes polarization toward Th17 [79]. More specifically, aldosterone induced DCs to secrete IL-6 and transforming growth factor-beta (TGF-β), which in turn activate CD8+T cells and promote CD4+T cells towards a Th17 phenotype [81]. Moreover, aldosterone downregulates the programmed death-ligand 1 (PD-L1) in DCs. The programmed death-ligand 1 is one of the ligands that suppress CD8+T cell activation, and this mechanism further amplifies CD8+T cell activation [82,83] (Figure 1).

A precise characterization of cardiovascular inflammation is extremely important to gain more insight into the pathophysiology of aldosterone-related end-organ damage. Indeed, plasma cytokine levels may represent a less-sensitive index of the underlying disease when compared to detailed immunophenotyping in the context of heart and vascular tissue. Therefore, a thorough description of immune cell populations in plasma and tissues might represent a more valuable approach to characterize chronic inflammation states that are dependent on the alteration of the MR/aldosterone pathway.

## 4. Aldosterone as a Novel Marker of Obesity

Excessive activation of MR in adipose tissue contributes to several metabolic alterations often observed in obesity and metabolic syndrome. Obesity is determined by an excess of adipose tissue, in order to store excess lipids and calories, which results in white adipose tissue (WAT) expansion through two possible mechanisms: increase in cell number (hyperplasia) and/or cell size (hypertrophy) [84]. In turn, dysfunctional adipocytes promote macrophage recruitment within WAT through the production of several chemokines (e.g., monocyte chemoattractant protein-1 (MCP-1) and IL-8) [85,86]. This then contributes to several obesity-related complications, in particular low-grade chronic inflammation, fat mass expansion and insulin resistance [87,88,89]. In obesity states, infiltrating macrophages undergo a polarization shift from an anti-inflammatory phenotype (M2) to a proinflammatory one (M1) [90]. Several studies have shown that MR activation triggers adipose tissue inflammation [91]. In particular, aldosterone determines an up-regulation of several proinflammatory adipokines (e.g., tumor necrosis factor alpha (TNFα), MCP-1, IL-6, and leptin) and reduces adiponectin expression. The mineralocorticoid receptor pharmacological blockade is able to reduce the total number of hypertrophic adipocytes in murine models of obesity, with a subsequent modification of adipocyte secretory capacity [92]. The mineralocorticoid receptor is also able to affect macrophages polarization. Indeed, aldosterone promotes a classic proinflammatory profile in human monocyte-derived macrophages (M1), whereas the MR antagonist eplerenone elicits a switch to the anti-inflammatory profile (M2) [93]. Moreover, aldosterone favors an increase in intracellular ROS levels in murine preadipocytes, whereas MR blockade reverses such increase and reduces ROS production in the adipose tissue of obese mice [92]. Finally, MR antagonism in mice fed a high-fat diet has been shown to improve glucose tolerance and to prevent white fat expansion and body weight gain [72,94]. Altogether, these data demonstrate that MR activity plays a relevant role in the pathogenesis of the chronic low-grade inflammatory state and adipocyte dysfunction observed in obesity [95].

Our research group has characterized the effects of aldosterone and MR on adipocytes. We first demonstrated that MR expression in murine preadipocytes gradually increases along differentiation, driving the acquisition of the mature adipocyte phenotype via increased expression of peroxisome proliferator-activated receptor-gamma (PPARγ) [96], the “master gene” of adipogenesis in mammals [97]. Pharmacological MR antagonism determines a marked antiadipogenic effect in both murine and human preadipocytes by decreasing the expression of PPARγ [98]. Accordingly, MR mRNA and protein have been both detected in human visceral adipose tissue (VAT) [99] and specific MR knockdown in primary human visceral preadipocytes significantly reduced PPARγ expression and disrupted adipose differentiation process [100]. Importantly, adipocyte MR expression is higher in obese subjects, as well as in VAT when compared with subcutaneous adipose tissue [101]. These findings strongly indicate that adipocyte MR is more abundant in obese subjects, and its excessive activation contributes to adipocyte hypertrophy and dysfunction, which are frequently observed in obesity states [11]. 

**Figure 2:**
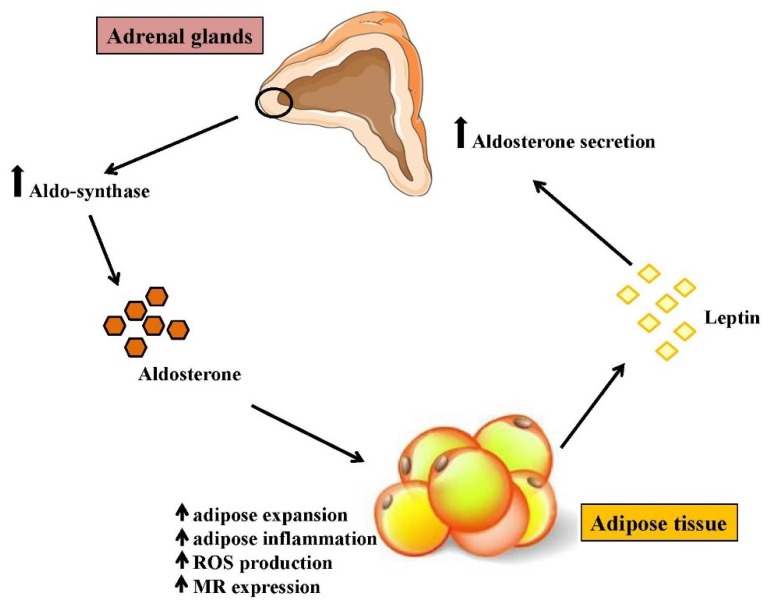
Cross-talk between adipose tissue and adrenocortical cell. Leptin secreted by adipose tissue stimulates aldosterone secretion from adrenal cortex increasing aldosterone synthase expression and aldosterone production in adrenal cells. Aldosterone in turn binds and activates MR at adipocyte level, favoring adipocyte differentiation, hypertrophy and inflammation. This vicious cycle leads to adipose tissue expansion and inflammation, reactive oxygen species (ROS) production and increase in MR expression.

Obesity and metabolic syndrome are strictly associated with an increased risk of cardiovascular disease, including left ventricular hypertrophy, coronary artery disease, hypertension, congestive heart failure, and vascular stiffness [102]. A large body of evidence indicates an important contribution of aldosterone/MR system into development of metabolic syndrome [103,104]. A higher prevalence of metabolic syndrome and increased cardiovascular events have been observed in patients affected by primary aldosteronism, when compared to essential hypertension [39,105]. Recently, Min et al. observed that aldosterone levels were higher in sera from patients with metabolic syndrome, and directly correlated to waist circumference, blood pressure and plasma triglycerides [106]. Obese subjects are characterized by high aldosterone and normal or low cortisol plasma levels [107,108]. This evidence supports the hypothesis that hyperaldosteronism and obesity could be linked by a mechanistic relationship, and the aldosterone/MR system may represent a mediator for cardiometabolic disease induced by obesity [108,109,110]. It is well established that unknown molecules secreted by adipose tissue are able to directly stimulate aldosterone production by the adrenal glands, and this effect is independent of renin-angiotensin-aldosterone system (RAAS) activation [111,112]. Recently, leptin has been proposed as one of the adipose tissue-derived products able to induce aldosterone synthesis. Huby et al. showed that leptin increases aldosterone synthase expression and function [113]. Leptin overexpression in obesity is able to directly stimulate the adrenals, leading to an increased production of aldosterone, which in turn binds and activates MR at the adipocyte level. Such a vicious cycle leads to adipose expansion, chronic inflammation, oxidative stress and subsequent increase of aldosterone-releasing factors production by adipocytes [110] (Figure 2). In consideration of these findings and in line with elevated aldosterone plasma levels observed in obese subjects [106], aldosterone plasma levels can be considered as a novel biomarker of obesity, as its secretion is correlated with WAT expansion and inflammatory state.

## 5. Mineralocorticoid Receptor Downstream Molecules: Novel Biomarkers of Cardiometabolic Diseases?

Cardiac remodeling secondary to hypertension is characterized by inflammation and fibrosis. It is considered as a major risk factor for cardiovascular morbidity and mortality, and represents a leading cause of chronic heart failure [114]. Cardiac remodeling begins with an inflammatory state, which promotes changes in ECM, resulting in myocardial fibrosis [115,116]. A dysregulated expression of metalloproteases (MMPs) determines an altered ECM remodeling during fibrosis. Activation of MMPs induces both the degradation of ECM structural components and the activation of growth factors able to promote inflammation [117]. Aldosterone has long been considered an important trigger for organ damage in hypertension [118]; in fact, its levels are increased in hypertensive patients and in spontaneously hypertensive rats (SHR) [119,120]. Mineralocorticoid receptor activation leads to cardiac inflammation and fibrosis [121] and podocyte injury: it is now clear that the proinflammatory effects of aldosterone are mediated by NLPR3 inflammasome at the level of podocytes [71]. Most importantly, MR antagonism reduces mortality and morbidity in clinical trials [53,122,123].

Given the central role of MR in the development of cardiometabolic disease, we focused on three MR downstream molecules that recently emerged as specific mediators of MR activation.

### 5.1. Neutrophil Gelatinase-Associated Lipocalin Protein

The neutrophil gelatinase-associated lipocalin protein (NGAL) has been identified as a novel MR target in the cardiovascular system [124]. The neutrophil gelatinase-associated lipocalin protein is a 25-kDa glycoprotein of the lipocalin superfamily [125] expressed by several cell types, including renal cells [126], ECs and SMCs [127,128], cardiomyocytes [124] and some immune cells subpopulations, such as neutrophils, macrophages, and DCs [128,129,130,131]. This protein is a marker of renal injury [132]. Elevated NGAL plasma levels have also been associated to increased mortality in patients with heart failure [133] independently from kidney dysfunction [134]. Accordingly, a recent study demonstrated that NGAL plays an important role in cardiovascular injury induced by aldosterone [135], and represents a mediator of cardiac inflammation and fibrosis in post myocardial infarction [136]. However, cell types involved in NGAL production in mineralocorticoid-induced organ damage have not yet been clearly determined. We previously discussed the role of immune cells MR expression in the progression of cardiometabolic diseases. Interestingly, elevated NGAL plasma levels have been detected in animal models in response to proinflammatory stimuli, as well as in patients affected by acute/chronic inflammatory states [137,138]. The secretion of NGAL by immune cells may play an important role in mediating mineralocorticoid-induced hypertension and cardiac injuries, since NGAL is a direct MR target [137,138]. In accordance, Buonafine et al. recently demonstrated that NGAL secretion by immune cells plays a pivotal role in mediating mineralocorticoid-induced cardiac injuries [139]. Mice lacking NGAL in their immune cells were protected against cardiac inflammation and fibrosis induced by nephrectomy-aldosterone (NAS) 200 μg/kg/day-salt 1% challenge [139]. In consideration of these data, NGAL could be an eligible biomarker in cardiovascular diseases due to altered mineralocorticoid activation, besides its well-known relevance as a biomarker of renal injury.

### 5.2. Galectin-3

Hyperaldosteronism worsens fibrosis through the increase in the production of several proinflammatory molecules [140]. Galectin-3 (Gal-3) is a 29–35-kDa protein, member of the β-galactoside-binding lectin family, and it is expressed in several cell types such as fibroblasts [141], ECs [142], and inflammatory cells [143]. Recent evidence shows that Gal-3 mediates aldosterone-induced vascular remodeling and cardiac fibrosis [144,145]. Hypertensive aldosterone salt-treated rats showed increased Gal-3 expression at both mRNA and protein levels in the heart. Cotreatment with spironolactone or modified citrus pectin (MCP), a Gal-3 inhibitor [146], abolished cardiac Gal-3 mRNA and protein up-regulation. Interestingly, cardiac hypertrophy and dysfunction were prevented by spironolactone or MCP co-treatment [145].

In addition, pharmacological blockade of Gal-3 prevents the aldosterone-induced increase in inflammatory markers and in MMP activities, indicating Gal-3 as a possible novel mediator in cardiac inflammation. In human cardiac fibroblasts, Gal-3 inhibition was able to prevent the increase in inflammatory and fibrotic markers (MMP activities, and ECM components) induced by aldosterone [147]. These observations suggest a major role of Gal-3 in mediating aldosterone-induced cardiac remodeling due to myocardial inflammation and fibrosis, which in turn determines the development of HF. Accordingly, clinical studies show increased levels of Gal-3 in patients with HF [148]. Moreover, plasma levels of Gal-3 are correlated with serum ECM markers, and Gal-3 represents a prognostic factor in patients affected by coronary artery disease, given its role in plaque destabilization [149].

Cardiac fibrosis is also associated to obesity. High fat diet-fed animals show cardiac hypertrophy, fibrosis and an increase in superoxide anion and proinflammatory molecules production [150]. Ex vivo studies showed that aldosterone-activated MR promotes adipocyte differentiation and secretion of proinflammatory adipokines and leptin [96,151]. In obese subjects, MR expression is increased when compared with lean individuals, which has been shown in several preclinical models of obesity and metabolic syndrome [92,95,152]. In line with this, Gal-3 inhibition was recently found to prevent adipose tissue remodeling in obesity [153]. Ectopic fat in obese individuals shares some functional features with visceral adipose tissue, including leptin secretion. Interestingly, leptin secreted by epicardial fat can exert its action directly on the heart since epicardial fat leans closely against the myocardium [154]. Leptin is directly involved in cardiac fibrosis, exerting prooxidant and profibrotic effects, inducing cardiomyocytes hypertrophy [155,156,157], and affecting collagen turnover, as observed in high fat diet-fed mice. Galectin-3 is expressed in many tissues, including the heart, and its circulating levels significantly increase in obesity [158,159,160]. Given its ability to stimulate collagen deposition and exacerbate proinflammatory states, Gal-3 could be involved in leptin-induced cardiac collagen derangement [143,159]. To address this hypothesis, Martinez-Martinez et al. evaluated fibrosis and oxidative stress in cardiomyocytes from high fat diet-fed rats, as well as in vitro proliferation of cardiac fibromyoblasts extracted from rat heart exposed to elevated leptin levels. They showed that collagen synthesis induced by leptin is partly mediated by the production of Gal-3 [141]. Therefore, also taking into consideration that its plasma levels are increased in primary aldosteronism and obesity [141,159,160,161], Gal-3 emerges as a novel circulating biomarker of cardiac damage and cardiometabolic disfunction due to MR activation.

### 5.3. Lipocalin-Like Prostaglandin D2 Synthase

Experimental and clinical studies have clearly demonstrated that excess aldosterone is a risk factor for type-2 diabetes mellitus and metabolic syndrome [39]. Interestingly, pharmacological MR antagonism improves glucose tolerance and reduces insulin resistance in murine models [92,94,95,152]. A mouse model selectively overexpressing MR in adipocytes (adipo-MR) displayed all the characteristics of metabolic syndrome [101]. Importantly, these mice showed an increase in lipocalin-like prostaglandin D2 synthase (PTGDS) mRNA expression in VAT.

The PTGDS is an enzyme involved in adipose tissue pathophysiology [162,163,164,165,166]. The increase in its expression is abolished in the presence of the MR antagonist spironolactone. In addition, the increase in PTGDS mRNA levels in VAT and subcutaneous adipose tissue (SAT) from genetically obese db/db mice are significantly correlated to increased MR mRNA levels from the same adipose depots. Moreover, upon aldosterone treatment, differentiated SW872 human adipocytes show increased expression of PTGDS mRNA levels, which is prevented by coincubation with spironolactone. Finally, in obese patients, VAT shows higher expression of PTGDS mRNA levels when compared to SAT, and again a positive correlation between PTGDS and MR mRNA levels is observed [101]. Altogether, these data suggest a direct control of MR in PTGDS transcription in adipocytes. Lipocalin-like prostaglandin D2 synthase emerges as a novel MR target in both mice and human adipocytes, and it might represent a novel tissutal marker of MR activation in adipocytes.

## 6. Conclusions

Recent evidence shows that dysregulation of the aldosterone/MR system is strictly associated with several pathological states that are characterized by high cardiometabolic risk and end-organ damage, particularly at the level of the heart, vessels, kidney and adipose tissue. Diseases associated with an altered function of the aldosterone/MR system, such as hypertension, diabetes, chronic kidney diseases, obesity, heart failure, are distinguished by elevated mortality and costs.

To date, there are no validated clinical biomarkers of the aldosterone/MR system other than plasma circulating levels of aldosterone itself, plasma renin activity and electrolytes, in particular potassium [167]. However, plasma electrolytes only represent an indirect marker of the RAAS status, and can be affected by several factors other than MR activation, such as plasma volume, salt intake, adrenergic tone, etc. Moreover, these readouts are not necessarily associated with organ damage, therefore they cannot be considered as a veritable signature of cardiometabolic diseases, fully able to identify high risk patients, eligible to intensive lifestyle or pharmacological intervention for cardiovascular protection.

Therefore, there is an unmet need for novel biomarkers that are able to detect the early stages of selective organ damage, mediated by the aldosterone-MR pathway.

An ideal diagnostic biomarker has to respect several criteria, such as a reasonable balance between cost and benefit, which favors a rapid and correct diagnosis, and should provide information on the patient health status [168].

Recently, the field of biomarkers has shifted from purely diagnostic aspects to risk stratification, therapeutic indications and prognosis. Novel biomarkers should preferably be involved in specific pathophysiological pathways, leading to the initiation or exacerbation of the disease. In this context, aldosterone-MR pathway has been carefully explored in the last years, due to its intimate connections with several comorbidities, and recent studies yielded potentially interesting novel biomarkers. Of course, more studies are deemed necessary to confirm their actual prognostic value, their ability to provide useful information on the patient health status beyond signs and symptoms or other already available techniques.

**Figure 3:**
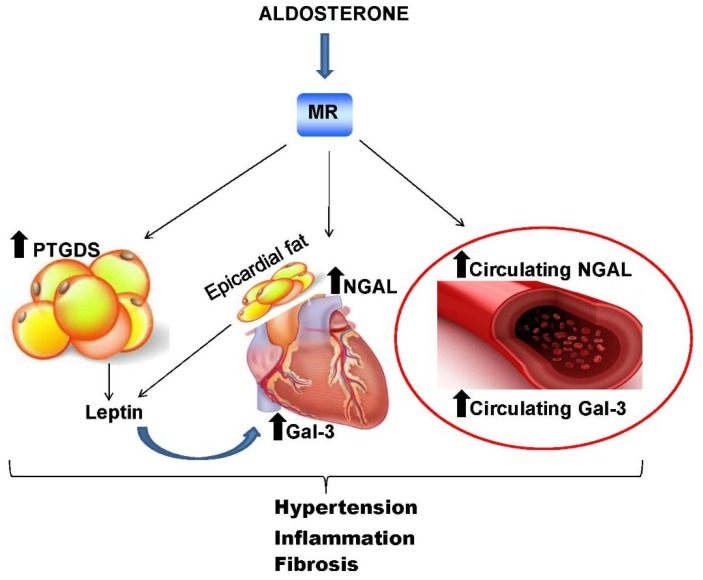
PTGDS, NGAL and Gal-3 as novel biomarkers in cardiovascular diseases induced by altered mineralocorticoid activation. MR activation by aldosterone induces the expression of different downstream molecules. PTGDS is expressed in adipose tissue, whereas NGAL and Gal-3 are expressed in the heart and vasculature; NGAL and Gal-3 are also detectable in the plasma. In obesity states, elevated leptin levels secreted by adipose tissue (in particular epicardial fat), directly activate heart MR, which in turn further promotes Gal-3 synthesis. All these molecules contribute to induce end-organ damage, through disarrangement of ECM and collagen. PTGDS: lipocalin-like prostaglandin D2 synthase; NGAL: neutrophil gelatinase-associated lipocalin; Gal-3: galectin-3.

Here, we have discussed the impact of potential novel biomarkers related to the aldosterone/MR system, which could help identify cardiovascular and metabolic detrimental conditions. Specifically, we focused on the effects of altered MR activation on distinct subpopulations of circulating and intra-tissue immune cells (Figure 1), and on MR downstream molecules (NGAL, Gal-3 and PTGDS), whose expression could represent a reliable biomarker of end-organ damage (Figure 3).
